# Surfactin induces maturation of dendritic cells *in vitro*

**DOI:** 10.1042/BSR20160204

**Published:** 2016-10-06

**Authors:** Wenwen Xu, Haofei Liu, Xiaoqing Wang, Qian Yang

**Affiliations:** * Key Lab of Animal Physiology and Biochemistry, Nanjing Agricultural University, Nanjing, 210095, People's Republic of China

**Keywords:** dendritic cells (DCs) maturation, nuclear factor-kappa B (NF-κB), surfactin

## Abstract

Surfactin has multiple immune activities, such as triggering immune-related defense responses and enhancing humoral and cellular immune responses. Although, the mechanisms are still unclear. The maturation of dendritic cells (DCs) is essential for inducing downstream immune response. To shed light on the mechanisms of surfactin-induced immune activities, we verified the influences of surfactin on DCs maturation. The results showed that after stimulated with 20 μg/ml surfactin for 24 h, DCs were conferred morphologic and phenotypic characteristics of a mature state, showing an increased shape index and up-regulated expressions of major histocompatibility complex II (MHCII) and CD40. Moreover, surfactin also induced DCs to release IL-6 and tumour necrosis factor-α (TNF-α), indicating that DCs were functionally mature. In addition, the IκB-α level in surfactin-treated DCs was significantly reduced whereas the nuclear p65 level was notably increased, preliminarily indicating that nuclear factor-kappa B (NF-κB) signalling pathway might play an important role in surfactin-induced DCs maturation.

## Abstract (precis)

This was the first report of which we were aware concerning the effects of surfactin on DCs maturation and NF-κB signalling pathway might be involved in this process.

## INTRODUCTION

Surfactin, a bacterial cyclic lipopeptide, is produced by various strains of *Bacillus subtilis* [1]. As one of the most effective biosurfactants, surfactin is built from a heptapeptide and a β-hydroxy fatty acid with variable chain lengths of 13–15 carbon atoms [2]. This biosurfactant is biodegradable and less toxic than chemical surfactants and has a lot of applicable potential in various fields [3]. A host of interesting features of surfactin has led to a wide range of applications such as inducing immune responses or serving as an adjuvant [4]. However, the mechanisms underlying surfactin-induced immune responses are still unclear.

Dendritic cells (DCs) are potent antigen presenting cells (APC) which play a critical role in the initiation and regulation of the immune responses [5]. DCs are derived from bone marrow progenitor cells and exist at two different stages: immature and mature [6]. *In vivo*, most DCs are immature ones, which have a strong ability to identify and capture inhaled substances. On the other hand, because of the low expressions of major histocompatibility complex II (MHCII) and co-stimulating factors, immature DCs are inability to initiate immune responses [7]. Uptake of antigens or certain stimulus would trigger maturation process in which DCs would turn to express high levels of MHCII and co-stimulating factors [8]. Mature DCs migrate to local draining lymph nodes, active naïve T-cells there, and initiate immune responses [9]. The maturity of DCs provides them with a tremendous ability of presenting antigens, occupying a momentous part in inducing downstream immune responses.

In this research, the effects of surfactin on DCs maturation were identified in the aspects of morphous, phenotypes, cytokines production, migration ability as well as the ability of stimulation on T-cell proliferation. Furthermore, whether nuclear factor-kappa B (NF-κB), an important signalling pathway of DCs maturation, was involved in this process was also detected. Our study aimed to gain a better understanding of the mechanisms for surfactin-induced immune responses.

## MATERIALS AND METHODS

### Surfactin and animals

Surfactin used in the present study was donated by Pro. Xuewen Gao from college of plant protection in Nanjing Agricultural University. Pro. Gao extracted it from *B. subtilis* G1 strain with methods followed previous studies [10,11]. The concentration of surfactin is no less than 95% detected by HPLC.

Male C57BL/6 and BALB/c mice, 4–6 weeks of age, were obtained from the Center for Comparative Medicine of Yangzhou University, China, and housed in plastic cages under standard specific-pathogen-free conditions following the University Ethics Committee's guidelines for at least 1 week before use.

### Generation of DCs

DCs were isolated and cultured with our advanced method [12]. In brief, bone marrow cells were obtained from femurs and tibias of wild-type male C57BL/6 mice and treated with red blood cells lysing buffer (Beyotime). The bone marrow cells were differentiated into DCs by being resuspended in complete medium RPMI-1640 (Invitrogen) supplemented with 10% fetal bovine serum, 1% penicillin–streptomycin, 10 ng/ml interleukin-4 (IL-4) and 10 ng/ml granulocyte-macrophage colony-stimulating factor (GM-CSF) (PeproTech) and plated at 1×10^6^ cells per ml in six-well plates. After 60 h of culture, the medium was gently discarded, and fresh medium was added. On day 6, non-adherent and loosely adherent cells were harvested and centrifuged to remove debris and dead cells, then transferred into six-well plates, and cultured overnight in complete medium. On day 7, only cultures with >90% cells expressing CD11c by FACS were used for the experiments.

### Cell viability assay by CCK-8 assay

The previously harvested DCs were plated at 2×10^5^ cells per ml in a 24-well plate and incubated with various concentration of surfactin (0.02, 0.2, 2 and 20 μg/ml) for 24 h. Then the cells of each well were collected and centrifuged (1500 rpm, 10 min, 4°C). The supernatant and sediment were separately added into a 96-well plate, 100 μl per well. Subsequently, 10 μl CCK-8 was added to each well and incubated for another 2 h at 37°C away from light. The extinction was measured at 450 nm using a Sunrise Microplate Reader (Tecan). The average attenuance formed in control cells was taken as 100% viability, and the results of treatments were expressed as a percentage of the control.

### Morphous assay by microscope observation

The previously harvested DCs were plated at 5×10^5^ cells per ml in a 12-well plate and separately incubated with PBS (0.01 M), lipopolysaccharide (LPS) (100 ng/ml) or surfactin (20 μg/ml) for 24 h. The morphous of DCs was observed by an optical microscope and the shape indexes were calculated. A DC shape index is identified to be the value of the length of its long axis divides that of its short axis.

### Phenotype assay by FACS

The previously harvested DCs were plated at 5×10^5^ cells per ml in a 12-well plate and separately incubated with PBS (0.01 M), LPS (100 ng/ml) or surfactin (20 μg/ml) for 24 h. Then the cells of each well were washed with cold PBS and stained with fluorescent mAbs specific for mouse CD11c, CD40 and MHCII, or the respective isotype controls at 4°C for 1 h as per the manufacture's guidelines. After washing three times with PBS, the cells were phenotypically analysed by FACS.

### Cytokine assay by enzyme-linked immunosorbent assay

The productions of cytokines interleukin-6 (IL-6) and tumour necrosis factor-α (TNF-α) were measured using enzyme-linked immunosorbent assay (Boster), and performed according to the manufacture's guidelines.

### Migration assay by transwell

Migration assay was performed as described previously [13]. DCs in serum-free medium were placed in a 12-well transwell migration chamber (pore size, 5 μm; Corning, NY, USA). 0.1 ml RPMI-1640 medium containing 1×10^5^ DCs was loaded on to the upper well. 0.6 ml RPMI-1640 medium containing C–C chemokine ligand 19 (CCL19) (200 ng/ml) was added to the lower chamber to induce cell chemotaxis. After incubation for 4 h at 37°C, the migrated cells were collected from the lower wells, and the number of CD11c^+^ cells was determined by FACS.

### Allogenic mixed lymphocyte reaction

The functional activity of DCs was reflected in the primary allogenic mixed lymphocyte reaction assay. T-cells were separated from allogenic BALB/c mice MLNs by a T-cell isolation kit (Miltenyi) and then labelled with CFSE (Invitrogen) according to the manufacturer's guidelines. T-cells as responder cells were implanted with DCs at 5×10^5^/well (DC/T-cell ratios of 1:1) in a 12-well plate for 5 days and then detected by FACS.

### Western blotting

The nuclear and cytoplasmic protein of DCs was extracted separately by a nuclear and cytoplasmic protein extraction kit (Beyotime) and its concentration was determined via BCA protein assay (Pierce). The normalized amounts of protein were denatured in SDS, electrophoresed on a SDS/12% polyacrylamide gel, and transferred to PVDF membranes. The PVDF were then blocked for 2 h with 5% skim milk in TBS Tween 20 (TBST) and then washed with TBST five times. Subsequently, the membranes were incubated with antibodies (IκB-α or p65) at 1:1000 dilutions with gentle shaking at 4°C overnight. After washing five times with TBST, the membranes were exposed to HRP-conjugated secondary antibody at 1:5000 dilutions for 2 h at the room temperature. After five washes with TBST, protein bands were visualized with enhanced chemiluminescence (ECL) Western blotting detection reagents (Pierce). The ECL image was recorded using the FluorChem Xplor (Alpha Innotech), and the attenuance of an equal surface area for each band was analysed with Quantity One (Bio-Rad Laboratories). All blots were stripped and reprobed with polyclonal anti-GAPDH antibody to as certainly equal loading of proteins.

### Statistical analysis

Results were expressed as means±S.D. One-way ANOVA was employed to determine statistical differences among multiple groups. *P* values < 0.05 were considered significant (*P<0.05, **P<0.01).

## RESULTS

### Generation of DCs and the toxicity analysis

**Figure 1:**
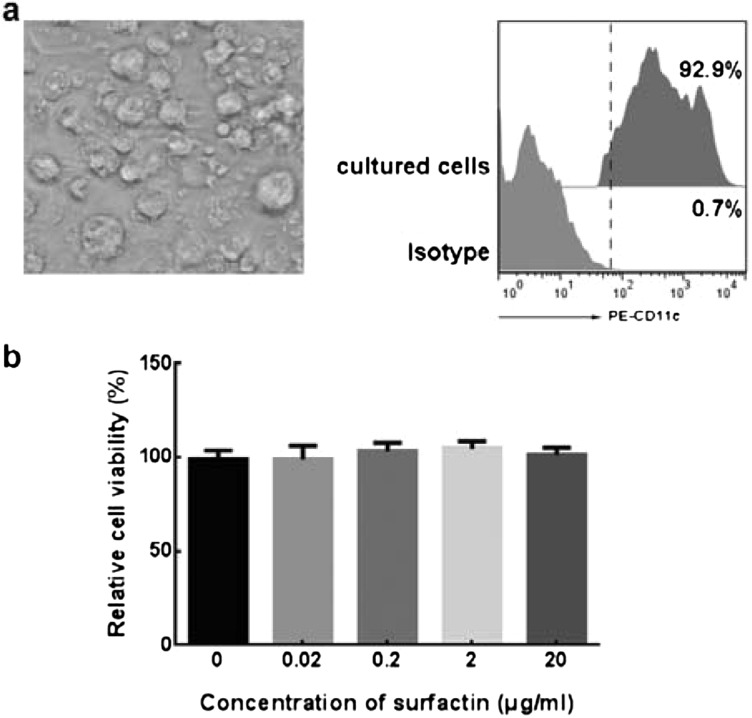
Identification of cultured cells and toxicity analysis of surfactin on DCs (**a**) Mouse bone marrow cells were obtained from femurs and tibias and cultured for 6 days. The cultured cells showed plenty of dendritic extensions (left) and high expression of CD11c (right). (**b**) Effect of surfactin on DCs survival based on CCK-8 assays. Cells were treated with different concentrations of surfactin for 24 h. Data were expressed as percentage reduction in CCK-8 and were calculated by dividing the attenuance (*D*) of corresponding supernatants from control wells (×100). The average *D* formed in control cells was taken as 100% viability. Results are representative of at least three independent experiments. Data are shown as mean±S.D.

DCs have a plenty of bifurcate prominence just like branches as their typical morphological characteristic. Mouse bone marrow cells were cultured in RPMI-1640 supplemented with IL-4 and GM-CSF for 6 days and their dendritic extensions were observed by an optical microscope. We found that after 6-day culture, mouse bone marrow cells showed a representative morphology of dendritic (Figure 1a left). Besides, given the CD11c is an essential surface marker on mouse DCs, its expression was detected by FACS to identify our cultures. As the result showed, more than 92% cells expressed CD11c, suggesting highly purified DCs were obtained (Figure 1a right). After that, the relative cell viability of DCs treated with different concentrations of surfactin (0.02, 0.2, 2 and 20 μg/ml) was assessed by CCK-8 assay. The result exhibited that all the concentration mentioned above had no obvious cytotoxicity effect on DCs (Figure 1b). For the sake of obtaining a visually significant outcome, the highest concentration of surfactin (20 μg/ml) was employed to the subsequent experiments.

### Surfactin-induced DCs maturation

**Figure 2:**
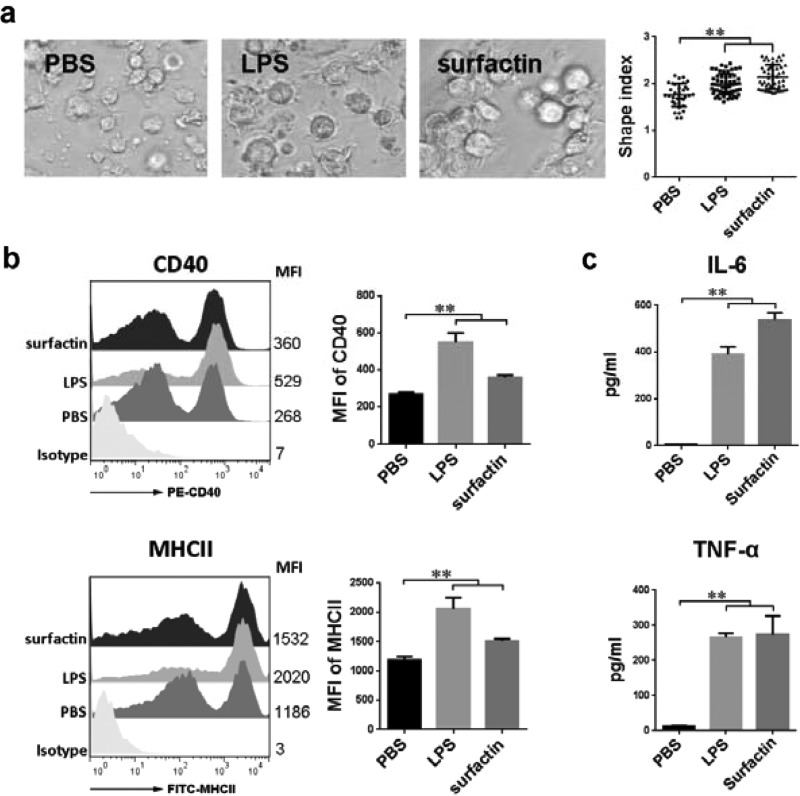
Surfactin-induced DCs maturation (**a**) DCs were treated with PBS, LPS or surfactin respectively for 24 h and the morphology of DCs dendrites were observed by microscopy. Scale bar=30 μm. The statistical result of cellular shape indexes is also showed. (**b**) DCs with different treatments were stained for the indicated surface molecules and analysed by FACS. The mean fluorescence intensity (MFI) was indicated in each histogram. The MFI values were shown as the mean±S.D. (**c**) Supernatants of DCs with different treatments were collected and tested for IL-6 and TNF-α by ELISA. Results are representative of at least three independent experiments. Data are shown as mean±S.D. **P<0.01.

Compared with immature DCs, mature DCs form longer extensions. To investigate the role of surfactin played on DCs maturation, the morphological change of DCs treated with surfactin was detected firstly. The result indicated that, same with LPS, a mature positive control, surfactin could also stimulate DCs maturation in terms of morphology (Figure 2a). Phenotypic change is also a marker of differentiation between mature and immature DCs. In addition, mature DCs usually possess high expressions of phenotypes, such as CD40 and MHCII. In our result, the expressions of both were significantly increased on DCs treated with surfactin, suggesting the positive effect of surfactin on DCs phenotypic maturation (Figure 2b). Furthermore, functionally mature DCs have an enhanced cytokines secretion. We assessed two representative cytokines, IL-6 and TNF-α secreted by DCs. The result showed that surfactin notably increased the production of these two cytokines (Figure 2c). In all, these results indicated that surfactin could induce morphological, phenotypical and functional maturation of DCs.

### Surfactin enhanced DCs migration ability

**Figure 3:**
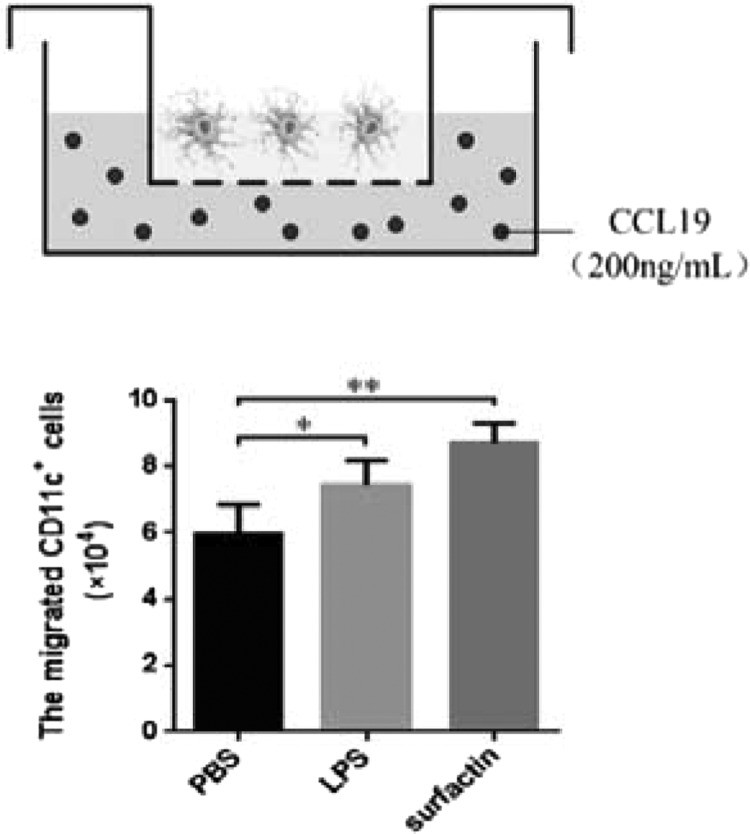
Surfactin enhanced DCs migration ability DCs were treated with PBS, LPS or surfactin respectively for 24 h, then added into the upper wells of a 24-well transwell chamber separately and CCL19 (200 ng/ml) was included in the lower wells. After 4 h, the number of DCs transferred to the lower wells was counted. Results are representative of at least three independent experiments. Data are shown as mean±S.D. *P<0.05; **P<0.01.

For mature DCs, migrating to lymphoid organs is a necessary step to induce downstream immune response. Mature DCs highly express C–C chemokine receptor type 7 (CCR7), conferring on them the ability to migration towards the concentration gradient of CCL19. The migration ability of DCs with different treatments was evaluated using chemotaxis assay in transwell chambers. As expected, the migration ability of surfactin-treated DCs was remarkably improved (Figure 3).

### Surfactin-treated DCs promoted T-cell proliferation

**Figure 4:**
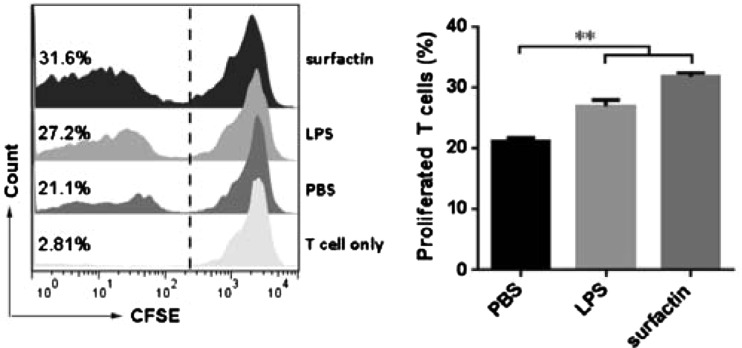
Surfactin-treated DCs promoted T-cell proliferation DCs were treated with PBS, LPS or surfactin respectively for 24 h, then co-cultured with CFSE-labelled T-cells at a ratio of 1:1. Five days later, T-cell proliferation was detected by FACS. Percentages stand for the proportion of T-cells that proliferated. Results are representative of at least three independent experiments. Data are shown as mean±S.D. **P<0.01.

Mature DCs could interact with T-cells and drive immune responses. So next we investigated the potential of surfactin to prime T-cells proliferation after its interaction with DCs. The allostimulatory capacity of surfactin-treated DCs was assessed by allogenic mixed lymphocyte reaction. The result indicated that DCs treated with surfactin had an increased ability to promote T-cell proliferation (Figure 4).

### Surfactin induced the activation of NF-κB on DCs

**Figure 5:**
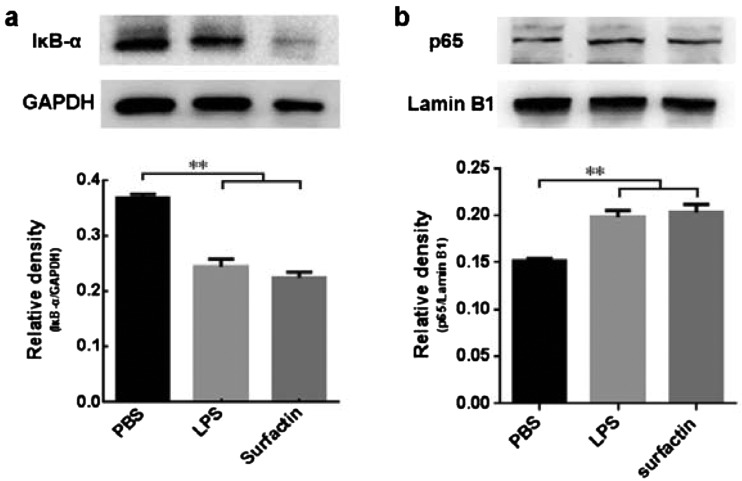
Surfactin induced the activation of NF-κB on DCs DCs were treated with PBS, LPS or surfactin respectively for 24 h, then the cytoplasmic and nuclear protein were extracted separately to detect IκB-α (**a**) and p65 (**b**) by Western blot. The quantifications of the blots are also shown. Results are representative of at least three independent experiments. Data are shown as mean±S.D. **P<0.01.

NF-κB activation is an important event underlying DCs maturation. On account of a conjecture whether NF-κB would be involved in surfactin-induced signalling pathways in DCs, the content of IκB-α and p65 were detected from DCs cytoplasmic and nuclear protein separately. We found that surfactin could promote the degradation of IκB-α and at the same time, increase the nuclear localization of p65, indicating that surfactin-induced DCs maturation was accompanied by the NF-κB activation (Figures 5a and 5b).

## DISCUSSION

This was the first report of which we were aware concerning the effects of surfactin on DCs maturation. As the most well-known biosurfactant produced by *B. subtilis*, surfactin has been reported to have multiple immune activities, such as, enhancing humoral and cellular immune responses [14], and triggering immune-related defense responses after intramuscularly or subcutaneously immunized [15]. Although, little is known about the mechanisms underlying surfactin-induced immune responses.

As the most notable professional APC, DCs are considered to play a crucial role in the establishment of both innate and adaptive immune responses [16], and their maturation is responsible for the ability to tailor immune responses [6]. DCs might serve as a potential target for surfactin to governing immune responses [17]. Focus was shifted on the possible involvement of surfactin in inducing DCs maturation, which is extremely important in regulating immune responses. Compared with immature DCs, mature ones have longer extends, express higher level of phenotypes, such as CD40 and MHCII, and produce more cytokines [18]. Our results showed that surfactin could induce DCs to undergo mature progress, characterized by highly expressing MHCII and CD40, and enhanced expressions of IL-6 and TNF-α. The ability to induce DCs maturation might provide the basis for surfactin to strengthen immune responses. Although, DCs maturation is necessary but not sufficient for inducing downstream immune responses. The expressions of CCR7 will confer DCs the ability to migrate towards draining lymph nodes [19], where have a high concentration of CCL19, and interact with T-cells and initiate immune responses [6]. In the present study, we found surfactin-induced DCs maturation combined with an enhanced migration ability towards CCL19, indicated that these DCs could arrive at draining lymph nodes more effectively. Moreover, we also investigated the ability of surfactin-treated DCs to promote allogeneic T-cell proliferation. As expect, surfactin enhanced the proliferation ability of DCs. Hence, surfactin-induced DCs maturation, enhanced their migration ability to draining lymph nodes, and promoted T-cells proliferation. These series of effects on DCs showed us a possible mechanism responsible for the immunological enhancement of surfactin.

NF-κB is one of the most classic signal transduction pathways in physiological processes and has been reported to be involved in the DC maturation [20]. In the resting state, IκB-α combines with two subunits, P65 and P50. After activation by upstream signals, IκB kinase ubiquitinates and phosphorylates, then degrades IκB-α. Consequently, the two subunits transferred into nuclear from plasma, leading to the expressions of a large number of target genes [21]. Previous studies on the involvement of the NF-κB system in DCs maturation marker expression and DCs functional properties showed differences depending on the stimulus employed [22]. In this sense, we addressed the role of the NF-κB signalling pathways in surfactin-induced DCs maturation. We demonstrated that surfactin induced the activation of NF-κB in DCs. NF-κB signal might involve in surfactin-induced DCs maturation.

To sum up, surfactin could induce immature DCs turn to a mature state, having an enhanced migration ability and proliferation promoting ability, and in this process, NF-κB was a signalling pathway involved. These findings disclosed the possible mechanism underlying surfactin-induced immune promoting effects.

## Abbreviations

APC: antigen presenting cells
CCL19: C–C chemokine ligand 19
CCR7: C–C chemokine receptor type 7
DC: dendritic cell
ECL: enhanced chemiluminescence
GM-CSF: granulocyte-macrophage colony-stimulating factor
IL-4: interleukin-4
IL-6: interleukin-6
LPS: lipopolysaccharide
MFI: mean fluorescence intensity
MHCII: major histocompatibility complex II
NF-κB: nuclear factor-kappa B
TBST: TBS Tween 20
TNF-α: tumour necrosis factor-α
